# The crystallization additive hexatungstotellurate promotes the crystallization of the HSP70 nucleotide binding domain into two different crystal forms

**DOI:** 10.1371/journal.pone.0199639

**Published:** 2018-06-27

**Authors:** Aengus Mac Sweeney, Alain Chambovey, Micha Wicki, Manon Müller, Nadia Artico, Roland Lange, Aleksandar Bijelic, Joscha Breibeck, Annette Rompel

**Affiliations:** 1 Drug Discovery Biology, Idorsia Pharmaceuticals Ltd., Allschwil, Switzerland; 2 University of Vienna, Faculty of Chemistry, Department of Biophysical Chemistry, Vienna, Austria; Weizmann Institute of Science, ISRAEL

## Abstract

The use of the tellurium-centered Anderson−Evans polyoxotungstate [TeW_6_O_24_]^6−^ (TEW) as a crystallization additive has been described. Here, we present the use of TEW as an additive in the crystallization screening of the nucleotide binding domain (NBD) of HSP70. Crystallization screening of the HSP70 NBD in the absence of TEW using a standard commercial screen resulted in a single crystal form. An identical crystallization screen of the HSP70 NBD in the presence of TEW resulted in both the “TEW free” crystal form and an additional crystal form with a different crystal packing. TEW binding was observed in both crystal forms, either as a well-defined molecule or in overlapping alternate positions suggesting translational disorder. The structures were solved by both molecular replacement and single wavelength anomalous diffraction (SAD) using the anomalous signal of a single bound molecule of TEW. This study adds one more example of TEW binding to a protein and influencing its crystallization behavior.

## Introduction

Growing crystals that are suitable for a detailed X-ray analysis of protein ligand interactions is a major bottleneck for many pharmaceutically relevant target proteins. The search for broadly applicable additives that enable protein crystallization is of central importance in addressing this bottleneck. The primary use of crystallization additives is for the generation or optimization of first protein crystals. However, as structure guided inhibitor optimization often requires crystal soaking systems with accessible ligand binding pockets, additives that generate alternative crystal packing arrangements are also of great value. The use of polyoxometalates (POMs), such as the tellurium-centered Anderson−Evans polyoxotungstate [TeW_6_O_24_]^6−^ (TEW), as crystallization additives has been demonstrated for three proteins including aurone synthase, tyrosinase and a new crystal form of lysozyme [1–4]), reviewed in [5–6].

**Fig 1:**
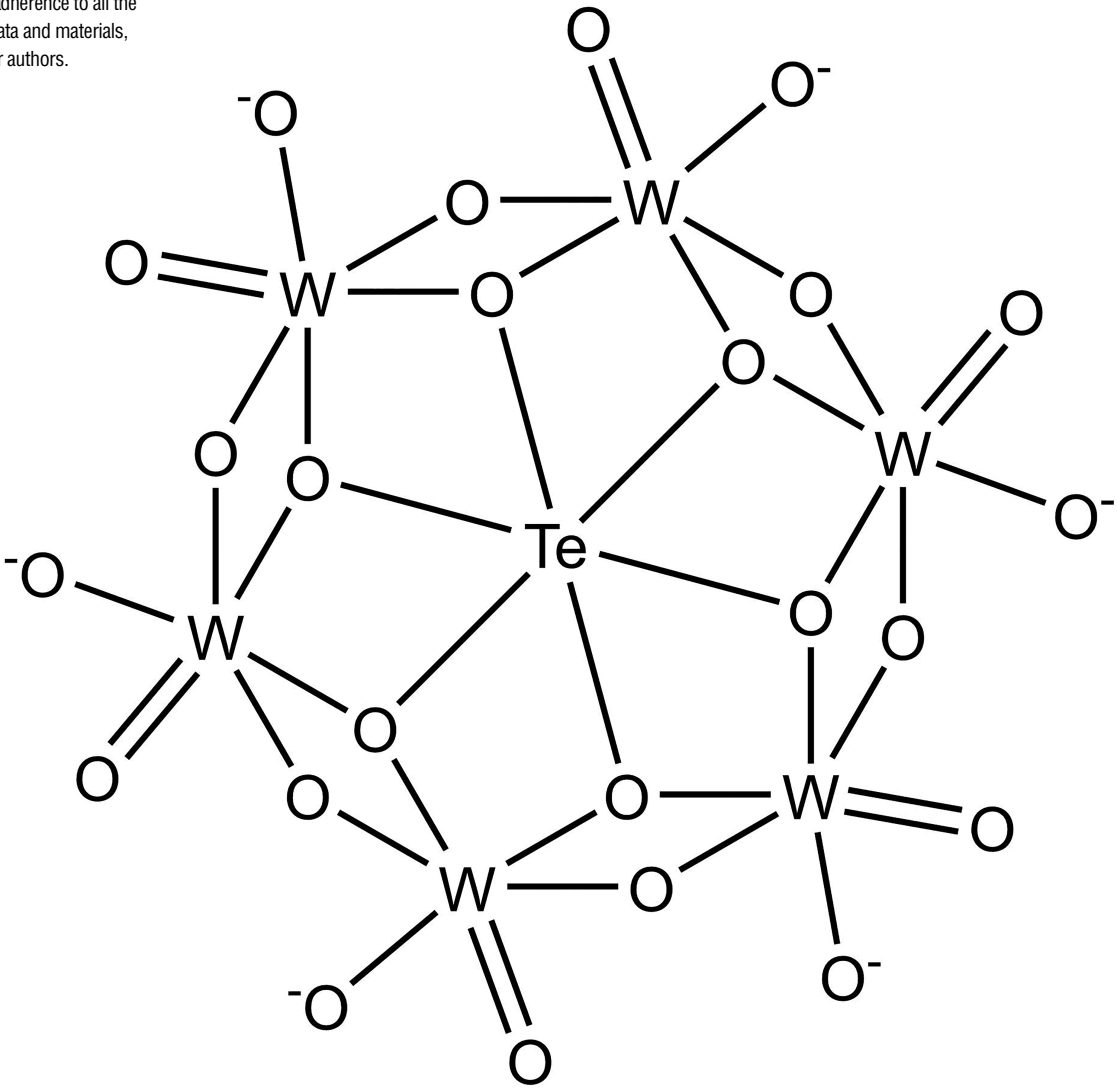
Chemical structure of TEW.

TEW is the best described member of the POM class of additives that are used to allow phasing and to promote protein crystallization. The effect of TEW on the crystallization behavior of proteins has been ascribed to both favorable entropic effects by displacing water molecules from protein surface patches upon binding [7] and the formation of new crystal contacts. The TEW molecule (Fig 1) has an approximately disk-shaped structure consisting of six edge-sharing WO_6_ octahedra enclosing an octahedrally arranged tellurium ion. Because of its high solubility and stability under most crystallization conditions, TEW has been used for the introduction of heavy and anomalously scattering atoms to solve the phasing problem (reviewed in [5]).

In order to test the effects of TEW on additional proteins, we screened the nucleotide binding domain (NBD) of heat shock protein 70 A1 (HSP70) in the presence of 10 mM TEW. The HSP70 family of proteins are ATP-dependent heat shock proteins that assist in protein folding and prevent the aggregation of newly synthesized peptides emerging from the ribosome. HSP70 is considered as a promising anticancer target as it is overexpressed by the majority of human tumors, and the expression of these proteins is typically a marker for poor prognosis. HSP70s consist of three structural domains: a 44 kDa amino-terminal ATPase domain followed by an 18 kDa substrate binding domain and a 10 kDa C-terminal domain, which forms a lid-like structure over the substrate-binding pocket that helps to trap the substrate. We chose HSP70 as under our standard crystallization conditions only a single crystal packing was observed. The crystal structures of two HSP70-TEW complexes are described, one of which was solved by single-wavelength anomalous diffraction (SAD) exploiting the anomalous signal of one bound TEW molecule.

## Methods

### HSP70 nucleotide binding domain expression

Codon optimized DNA encoding amino acids 1–382 of HSP70 1A (Uniprot P0DMV8) was synthesized (Geneart), cloned into a pET-28a vector and transformed into *E*. *coli* BL21(DE3) cells (Life Technologies / Thermo Fisher Scientific). Expression was carried out in shake flasks at 310 K. Cells were harvested 5 hours post induction with 1 mM IPTG. The cell pellet was resuspended in lysis buffer containing lysozyme and protease inhibitors. The cells were lysed using three freeze-thaw cycles followed by sonication. Insoluble matter was removed by centrifugation (30 min, 20,000 g, 277 K) and the supernatant was filtered. The filtrate was applied to a HiTrap SP cation exchange column and eluted with a step gradient of 5% / 70% / 100% elution buffer (20 mM MES, 10% glycerol, 1 M NaCl, pH 6.0). Fractions containing HSP70 were pooled and concentrated using a 30 kDa molecular weight cutoff membrane. The concentrated protein was further purified by size exclusion chromatography using a HiLoad 16/60 Superdex 200 column. Fractions containing HSP70 were pooled and concentrated to 15.4 mg/ml (0.34 mM) in 20 mM sodium/potassium phosphate, 300 mM potassium chloride, 0.5 mM TCEP, 1 mM magnesium chloride, pH 7.5. TEW was added to the protein at a final concentration of 10 mM, using a 100 mM stock solution of TEW (sodium salt, Na_6_TeW_6_O_24_) in water. The solution was incubated overnight for convenience (a shorter incubation would most likely suffice as binding of TEW is expected to occur very rapidly). ADP was added to the protein/TEW solution at a final concentration of 0.34 mM (equimolar to HSP70) 30 minutes prior to crystallization screening. ADP promotes HSP70 crystallization by binding to the ATP binding pocket and stabilizing the HSP70 structure.

### Crystallization

Crystallization screening of HSP70 in the absence and presence of TEW was carried out at 293 K using the sitting drop method with 0.3 μl each of protein and reservoir solution in Intelli-plates (Art Robbins). The Index screen (Hampton Research) was screened using an Oryx8 screening robot (Douglas Instruments). In the presence of TEW, protein crystals grew in multiple crystallization conditions, including 0.2 M magnesium chloride, 0.1 M Tris pH 8.5, 25% (w/v) polyethylene glycol 3,350 (crystal form 1) and 0.2 M sodium chloride, 0.1 M Bis-Tris pH 6.5, 25% (w/v) polyethylene glycol 3,350 (crystal form 2). In the absence of TEW, only crystals of crystal form 1 grew in multiple PEG conditions, however, crystal form 2 was only observed in the presence of TEW. All suitable crystals (1–2 per crystallization condition) grown in the absence or presence of TEW were stored for X-ray data collection to allow space group determination and an estimation of the diffraction quality.

### Data collection and processing

Crystals were mounted in nylon loops and cryocooled directly in liquid nitrogen. Diffraction data were collected at beamline PXIII of the Swiss Light Source (SLS). The diffraction data were processed using XDS [8]. The low overall completeness of the data from crystal form 1 is due to ice rings at 1.9, 2.2, 3.7 and 3.9 Å. Data in these regions were excluded during data processing.

### Structure solution by molecular replacement and SAD phasing

The structures were solved by molecular replacement using Phaser [9] and the published structure of HSP70 nucleotide binding domain (PDB entry 1S3X) [10]. In order to investigate the power of TEW as a phasing tool, SAD phasing of HSP70 crystal form 1 was carried out with the same dataset that was used for phasing by molecular replacement. The positions of the tungsten atoms were determined using the CCP4 software Crank2 and Prasa [11, 12] with the minimum distance between heavy atoms set to 3 Å and default values for all other settings. The TEW structure was not defined during the heavy atom search. Five of the six tungsten atoms as well as the central tellurium atom were within the top seven of identified peaks, allowing a clear identification of the TEW molecule by shape. Using the complete TEW molecule for initial phasing, density modification and automated building were carried out using Parrot and Buccaneer, respectively [13]. CCP4 software was run using the CCP4i2 interface [14].

Refmac5 [15] was used for refinement and building was carried out using Coot [16]. The structure of HSP70 from crystal form 1 was refined with anisotropic B-factors for all atoms, while the lower resolution structure from crystal form 2 was refined with isotropic B-factors for all atoms but the TEW molecules, which were anisotropically refined. Pymol [17] was used for preparation of the figures and APBS [18] for surface electrostatics calculation. The data processing and structure refinement statistics are summarized in Table 1. The structure models and crystallographic data are deposited in the PDB as entries 6G3R (crystal form 1) and 6G3S (crystal form 2).

**Table 1:** Data collection and refinement statistics.

	Crystal Form 1	Crystal Form 2
**Data collection**		
PDB entry ID	6G3R	6G3S
Space group	P 2_1_2_1_2_1_	P 2_1_2_1_2_1_
Cell dimensions		
*a*, *b*, *c* (Å)	46.4, 64.7, 143.4	69.9, 71.0, 99.3
Resolution (Å)^a^	48.0–1.40 (1.49–1.40)	44.5–2.30 (2.44–2.30)
*R*_merge_^b^	7.2 (80.2)	7.4 (50.4)
*I*/σ(*I)*	10.1 (1.3)	10.9 (1.9)
Completeness (%)	92.4 (95.8)	97.0 (97.8)
Multiplicity	5.0 (5.0)	3.4 (3.3)
*CC*_1/2_ (%)^c^	99.8 (77.7)	99.8 (85.0)
**Refinement**		
Resolution (Å)	48.0–1.40	44.5–2.30
No. reflections	75412	21023
*R*_work/_ *R*_free_	14.0 / 17.8	21.5 / 25.0
No. of HSP70 monomers	1	1
No. of TEW monomers No. of atoms	1	2
Protein	3026	2978
ADP, TEW and ions	66	160
Water	319	12
B-factors		
Protein	22.3	43.7
ADP, TEW and ions	24.8	80.7 (TEW1 57.7, TEW2 131.2)
Water	32.0	29.4
R.m.s Z		
Bond lengths (Å)	1.52	0.76
Bond angles (°)	1.33	0.86
Molprobity scores		
Clashscore	2	2
Ramachandran outliers (%)	0	0.5
Sidechain outliers (%)	0	3.3
RSRZ outliers (%)	0.5	2.4

^a^ Highest resolution shell is shown in parentheses

^b^ R_merge_ = Σ | I − <I> | / Σ I, where I is the observed intensity for a reflection and <I> is the average intensity obtained from multiple observations of symmetry-related reflections. R_free_ values are calculated based on 5% randomly selected reflections.

^c^ The mean intensity correlation coefficient of half-datasets [19]

### Measurement of HSP70 thermal stability by differential scanning fluorimetry

Differential scanning fluorimetry (DSF, also known as thermal shift) measurements were conducted to study the impact of TEW on the HSP70 protein stability. Experimental conditions e.g. protein/SYPRO orange ratio were optimized as recommended [20–23]. Assay ingredients and ligands were combined to obtain a mixture composed of HSP70 (8 μM), SYPRO Orange (10x in 20 mM Na/K phosphate pH 7.5, 300 mM KCl, 1 mM MgCl_2_, and 0.5 mM TCEP) and transferred to wells of PCR Plates (Micro Amp Fast 96-well Reaction Plates, Applied Biosystems). DSF was carried out with a StepOne PlusTM Real time PCR system. Thermal denaturation of HSP70 was monitored along 298 K to 328 K with a ramp rate of 1K/min. Protein melting curves were evaluated using the Thermal Shift Software (Applied Biosystems) to derive differences in midpoint melting-temperatures for HSP70 with and without ligands. TEW was tested at concentrations of up to 2 mM.

## Results

Crystallization of ADP-containing HSP70 alone (in the absence of TEW) resulted in crystals belonging to space group P2_1_2_1_2_1_ exhibiting the crystal packing referred to as crystal form 1 in the following. Crystals from all eight observed conditions were tested regarding their diffraction power and showed a similar range of diffraction quality as the crystals grown in the presence of TEW (1.4 to 1.8 Å). However, crystallization in the presence of 10 mM TEW resulted in both the original crystal form 1 (as observed without TEW) and a new crystal form, which was found to belong to the same space group but with significantly different unit cell dimensions (see Table 1). This crystal will be referred to as crystal form 2 in the following. For crystals grown in the presence of TEW, binding of TEW was observed.

Both crystal forms 1 and 2 have been previously published [24–26]. Crystal form 1 is the most frequent form of the HSP70 NBD in the PDB (entries 2E8A, 2E88, 3ATU, 3ATV, 3AY9 and 3JXU). All of these structures consist of the HSP70 NBD alone or in complex with ADP or AMP-PNP, without additional proteins or inhibitors. The only example of crystal form 2 (PDB ID 1HJO) [26] is a structure of the HSP70 NBD formed by co-crystallization with the poorly hydrolyzed ATP analog ATP-γ-S. Crystal form 2 was not observed in our previous crystallization experiments that were performed to determine the structures of five different HSP70-ligand complexes. Binding of TEW did not induce conformational changes of HSP70 in either crystal form (S5 Fig).

### Crystal form 1

**Fig 2:**
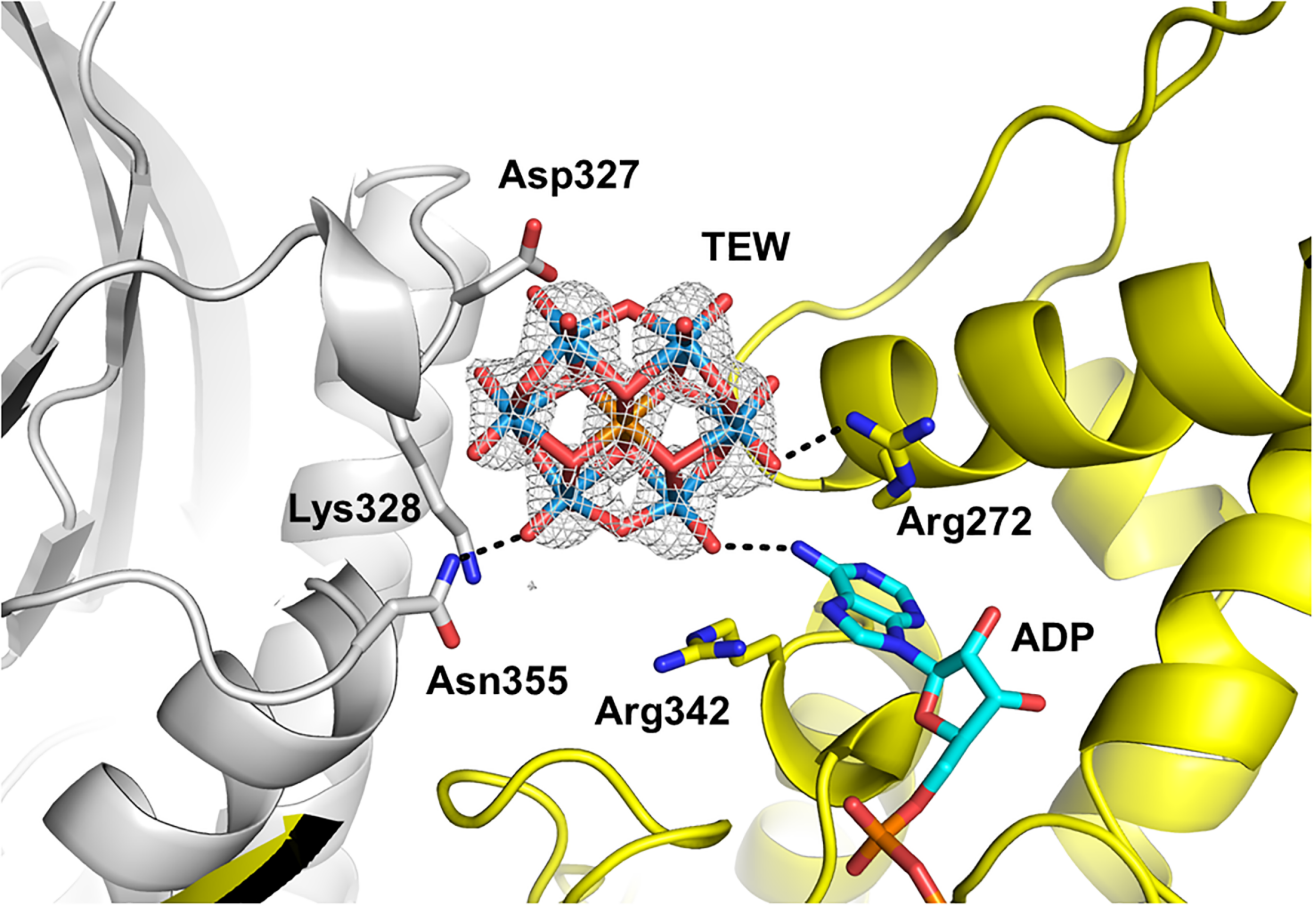
TEW bound to HSP70 at a crystal contact (crystal form 1). HSP70 is colored grey, the symmetry related HSP70 is colored yellow, with its bound ADP colored cyan. 2Fo-Fc electron density (contoured at 2 σ) is shown for the TEW molecule.

In crystal form 1, the asymmetric unit contains a single molecule each of TEW and HSP70 NBD. The crystal packing is identical to that of the crystals that were obtained in the absence of TEW. As observed in the absence of TEW, ADP is bound deeply in the ATP binding pocket. TEW is bound with an occupancy of 0.6 next to Asn355 (Fig 2). Terminal oxygen atoms of TEW form hydrogen bond interactions with the side chain nitrogen of Asn355 (2.7 Å) and the backbone nitrogen of Ala329 (2.7 Å). TEW lies at a crystal contact and interacts with the neighboring HSP70 molecule: a bridging O of TEW forms a water mediated interaction with the side chain of Ser276 (2.6 Å) and a terminal O of TEW forms a hydrogen bond with the amino group of the bound ADP (3.1 Å). Additional electron density around TEW suggests some disorder of this molecule in the structure. Additional water mediated hydrogen bond interactions may exist between TEW and the Asn355 side chain as well as the backbone carbonyl of Arg272 of a symmetry related monomer. The disorder/mobility of the TEW molecule prevented a more detailed analysis of the binding interactions, which was also observed in previous studies [2]. The TEW molecule is not located on a symmetry axis. The omit electron density map of TEW is shown in S1 Fig.

### Crystal form 2

In crystal form 2, TEW binds at two locations at the HSP70 surface exhibiting alternate and overlapping positions at both sites. In each case these alternate positions are related by a translation in the plane of the TEW molecule. Neither of the TEW sites are located on a crystallographic symmetry axis.

#### TEW site 1

**Fig 3:**
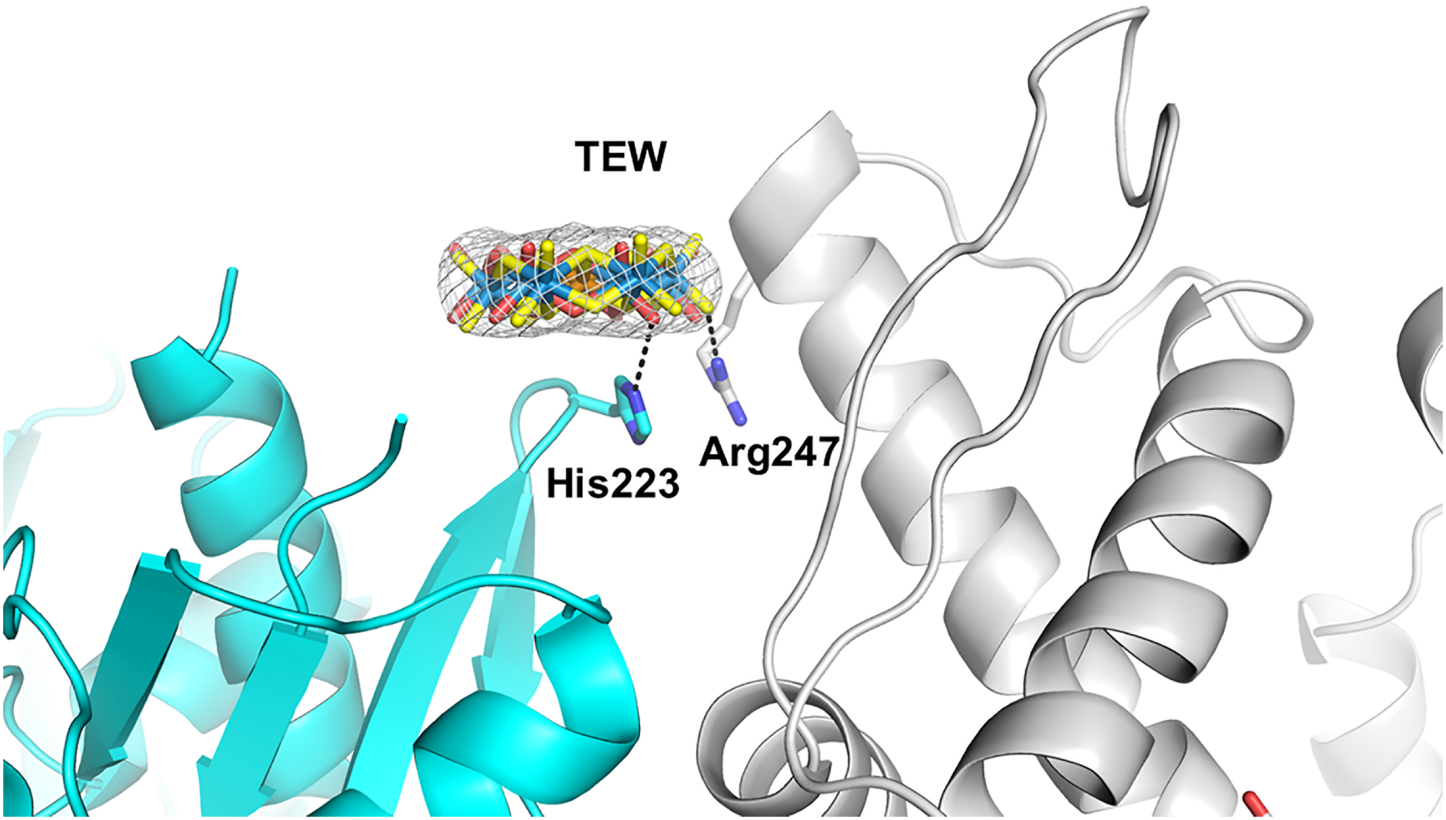
TEW bound to HSP70 at a crystal contact (crystal form 2, site 1). HSP70 is colored cyan, the symmetry related HSP70 is colored grey. The two alternate positions are shown with oxygen atoms colored red or yellow. 2Fo-Fc electron density (contoured at 2 σ) is shown for the TEW molecule.

Two alternate positions (referred to as A and B, each with an occupancy of 0.5) of the TEW molecule were observed, separated by a translation of 1.7 Å in plane of TEW (Fig 3). A nitrogen of the imidazole side chain of His23 interacts with the bridging oxygen atoms of TEW in both TEW positions, (with O12 in location A and with O13 in position B). TEW is located at a crystal contact and in both locations of TEW its terminal oxygen O21 forms multiple interactions with Arg247 of the symmetry related monomer, which has two side chain conformations. The omit electron density map of TEW is shown in S2 Fig.

#### TEW site 2

**Fig 4:**
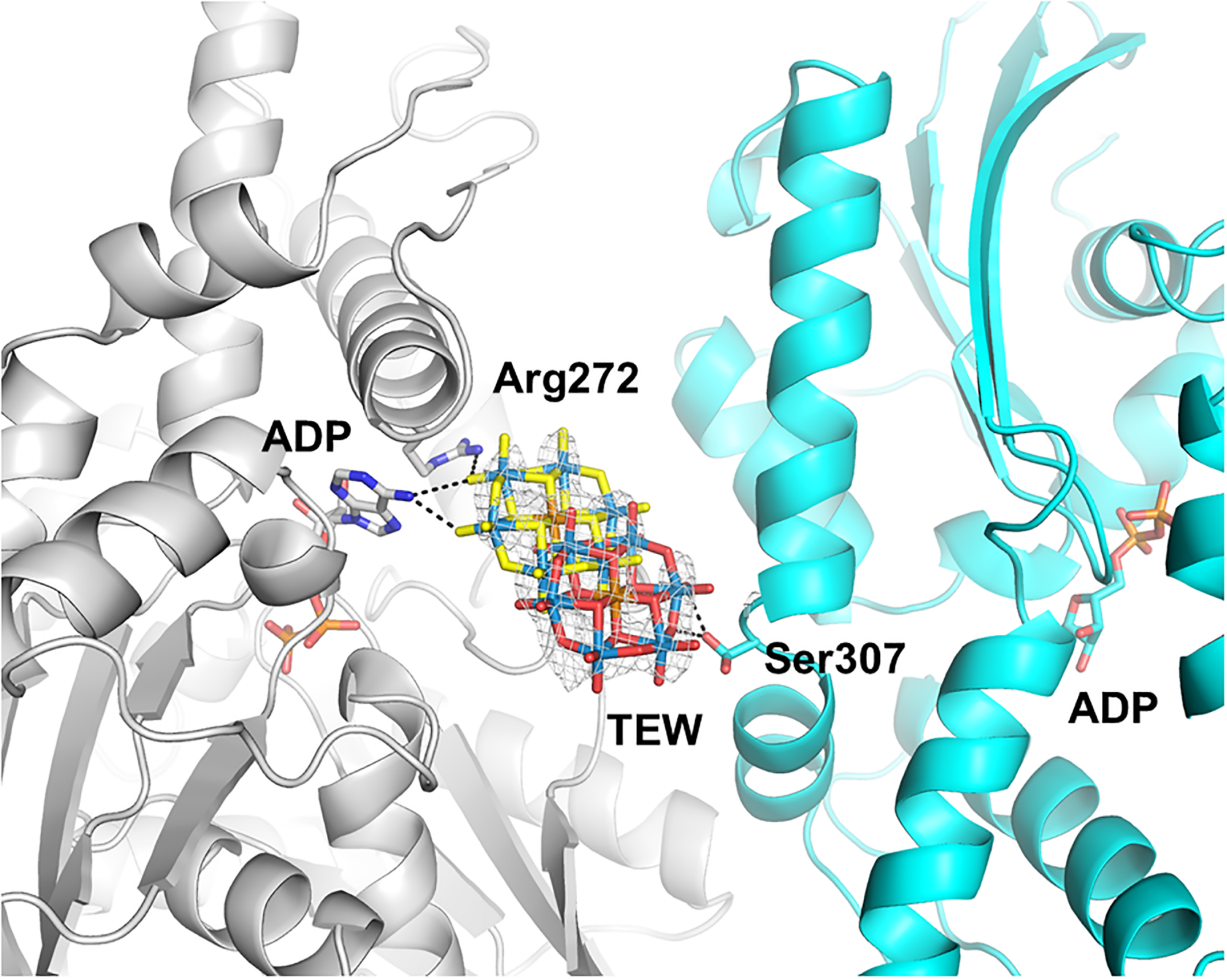
TEW bound to HSP70 at a crystal contact (crystal form 2, site 2). HSP70 is shown in cyan, the symmetry related HSP70 and its bound ADP are shown in grey. The two alternate positions of TEW are shown with the oxygen atoms colored in red (position A) or yellow (position B). 2Fo-Fc electron density (contoured at 1.5 σ) is shown for the TEW molecule.

Two alternate positions of TEW were tentatively modeled into the additional density near the amino group of the bound ADP, each with an occupancy of 0.3. Despite refinement with low occupancy, the average B-factor was very high (131.2 Å^2^) indicating high flexibility/mobility of TEW at this position. The two alternate positions of the TEW are related by a translation of 4.8 Å, again in the plane of TEW (Fig 4). The location of TEW is too poorly defined (even when using anomalous electron density maps, S3 Fig) to describe the binding interactions with confidence. TEW in position A is within hydrogen bond distance of the bound ADP (2.9 Å) and Asn364 (3.7 Å), while in position B it lies 3.7 Å from the hydroxyl group of Ser307 (3.1 Å) of the neighboring HSP70 molecule. In each position, the TEW molecule of site 2 interacts with only one molecule of HSP70. Therefore, this TEW could only act as a bridging molecule between two symmetry-related HSP70 molecules by translating rapidly between each HSP70 molecule explaining the high B-factor and distorted electron density. However, as we cannot distinguish static from dynamic disorder in the crystal structure, this question remains open.

**Fig 5:**
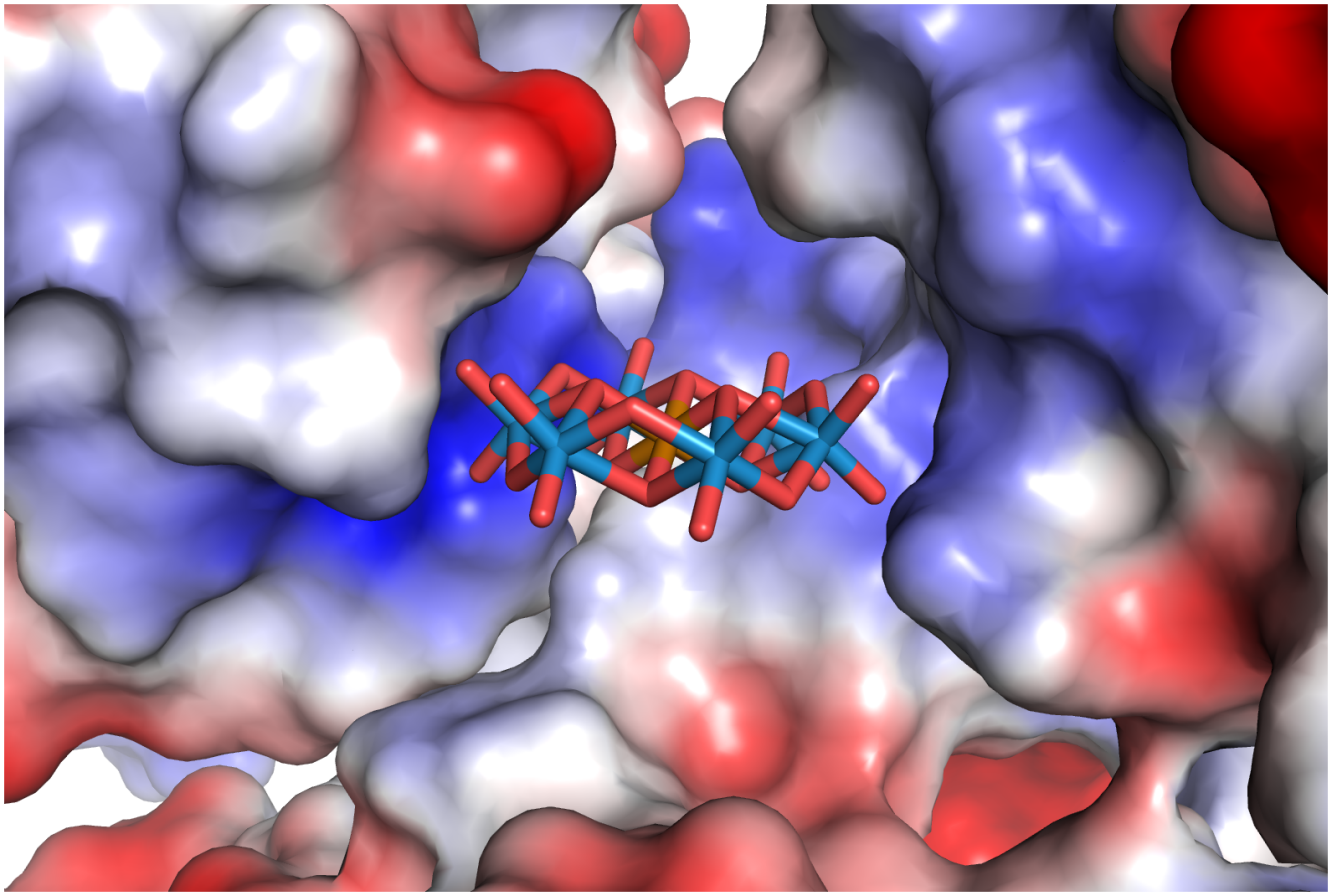
Surface potential of crystal form 1 calculated using APBS.

**Fig 6:**
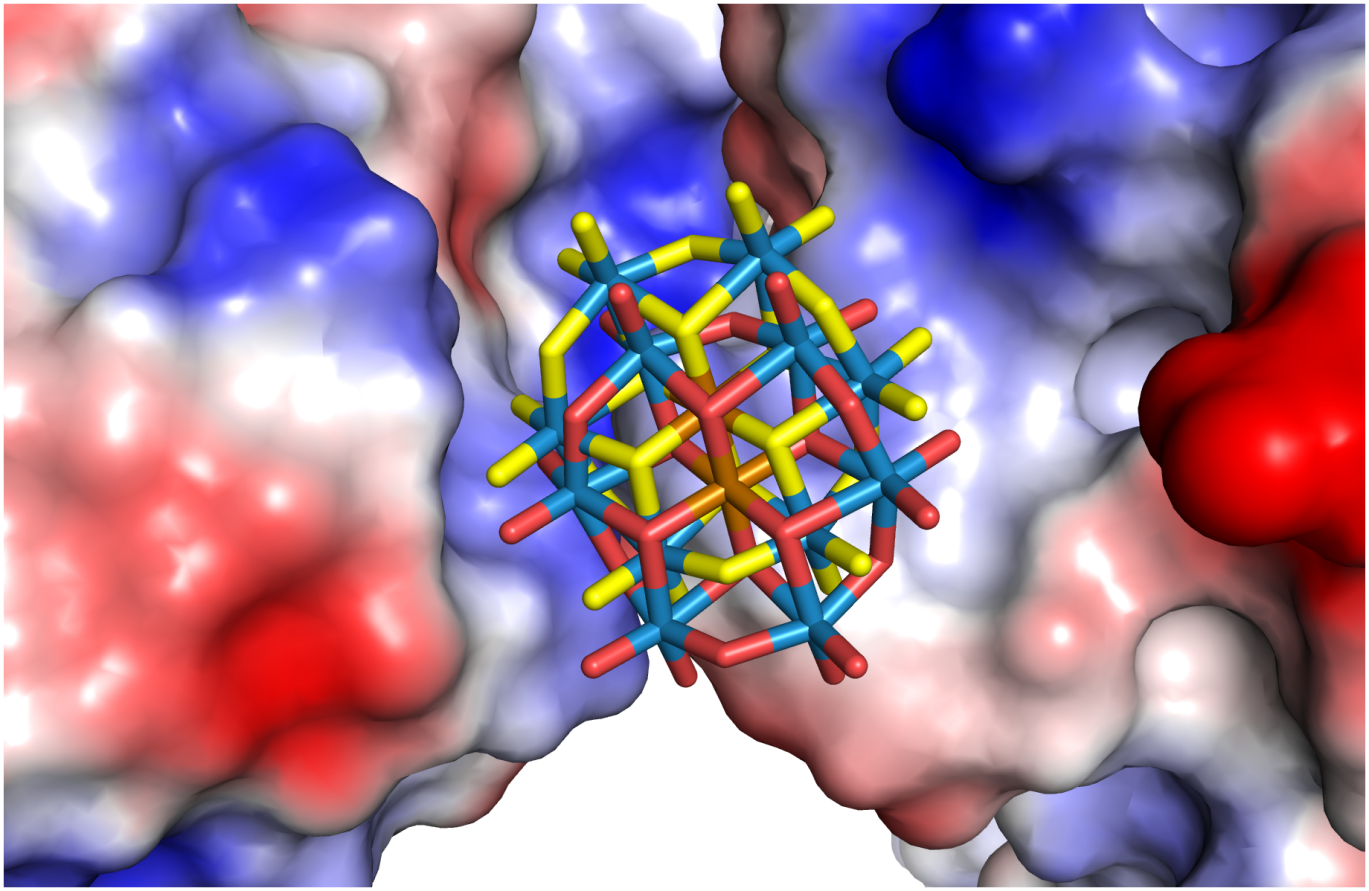
Surface potential of crystal form 2, site 1 calculated using APBS.

**Fig 7:**
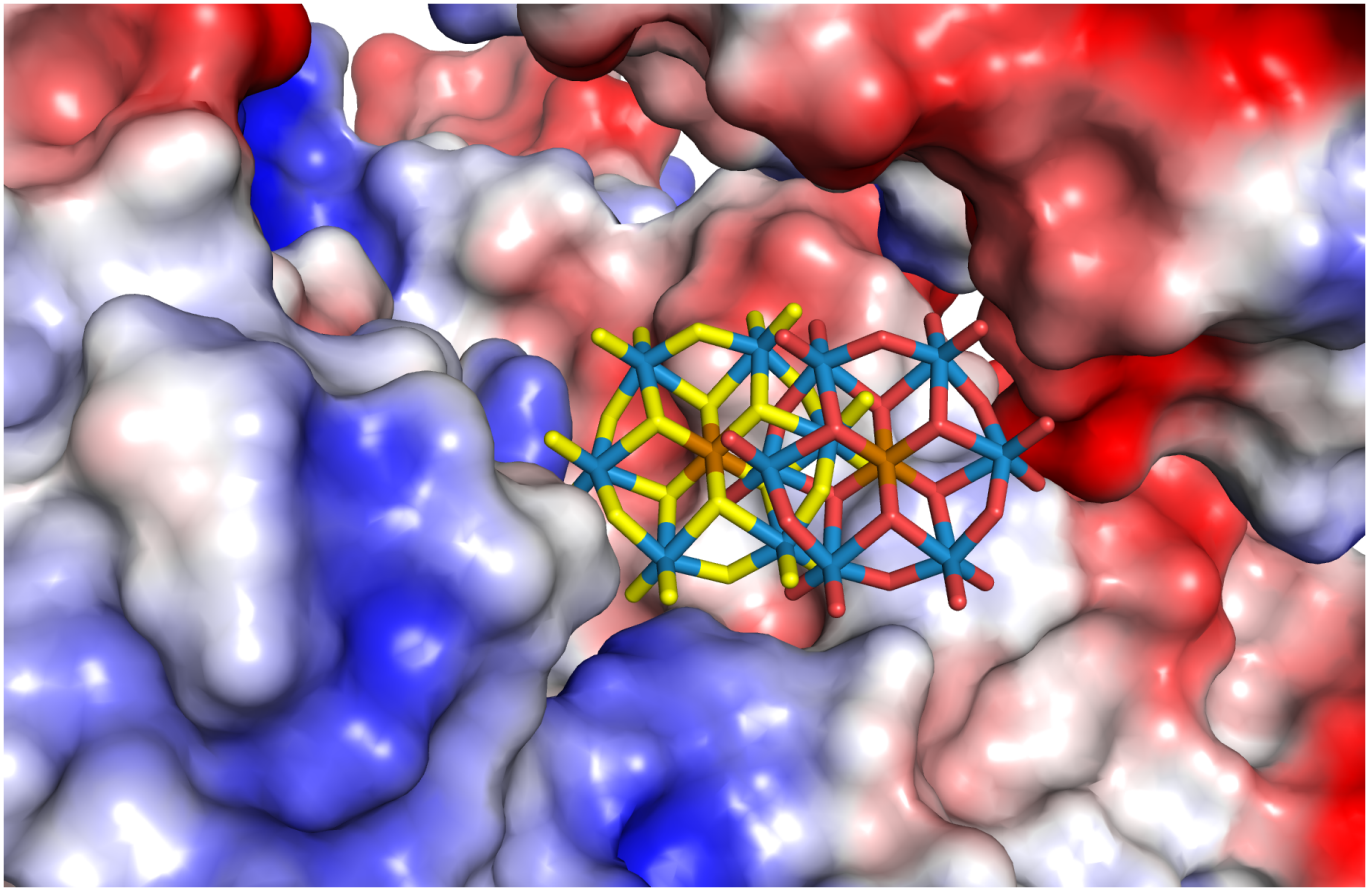
Surface potential of crystal form 2, site 2 calculated using APBS.

TEW crystal form 2 has three crystal packing interfaces with buried protein surface areas of 658, 416 and 217 Å^2^ [27]. Both TEW molecules are bound at the 416 Å^2^ interface between the protein and the symmetry related neighbor that is formed by the symmetry operation (-x, y+0.5, -z+0.5)(S6 Fig). This packing interface is present only in crystal form 2. It is possible that within the set of crystallization conditions we tested, TEW tips the balance in favor of crystal form 2 by stabilizing this interface. In each crystal form, the TEW molecule masks a positively charged patch on the HSP70 surface that is formed by Arg272, Arg342 and the amino group of ADP. Based on the electrostatic surface characteristics (Figs 5–7), TEW appears to bind preferentially at sites that offer a neutral surface towards the TEW “face” and a polar surface towards the TEW edge. This is in line with the observation that polyoxometalates display an unexpectedly strong tendency to bind to both polar and neutral surfaces [28]. A preference for binding to positively charged regions is in line with the net negative charge of -6. The large size and disk-like shape (approximately 9 x 9 x 3 Å^3^) (3) of TEW allows binding within the shallow clefts adjacent to protein crystal contacts, while its rigidity eliminates the direct entropic penalty associated with binding and immobilization of flexible ligands. Omit electron density maps of TEW are shown in S1–S4 Figs.

### SAD phasing of HSP70

The crystallization experiments were carried out mainly to observe the influence of TEW on HSP70 crystallization and the binding of TEW on the protein surface. As TEW is known to allow SAD phasing of novel protein structures, the structure of HSP70 was also solved by SAD phasing applying the same dataset which was used for molecular replacement of crystal form 1. Despite the presence of ice rings, the single bound TEW molecule was located and used for initial phasing. Automated building of the HSP70 structure was possible and resulted in a model containing 95% of the HSP70 residues, with an R-factor of 33.2% and an R-free of 35.2%. Only the additional electron density for the bound ADP (not added during automated building) resulted in some local errors in the structure.

### Measurement of HSP70 thermal stability by DSF

The interaction of TEW with HSP70 was studied by a fluorescence-based protein thermal-shift assay (Thermofluor), which is also referred to as differential scanning fluorimetry. DSF measures the stability of a protein and the concomitant enhancement or deterioration of its thermal stability upon ligand binding or changes in the physicochemical environment conferred by additives. The thermal denaturation process is recorded as an increase in fluorescence intensity of the environment sensitive fluorophore SYPRO orange which binds to hydrophobic sites that are exposed upon protein unfolding. The extent of a protein thermal shift induced by a ligand/additive reported as difference in midpoint melting temperature Tm with and without ligand/additive depends on concentration, affinity and molecular mode of interaction with the protein. [21, 29]

**Fig 8:**
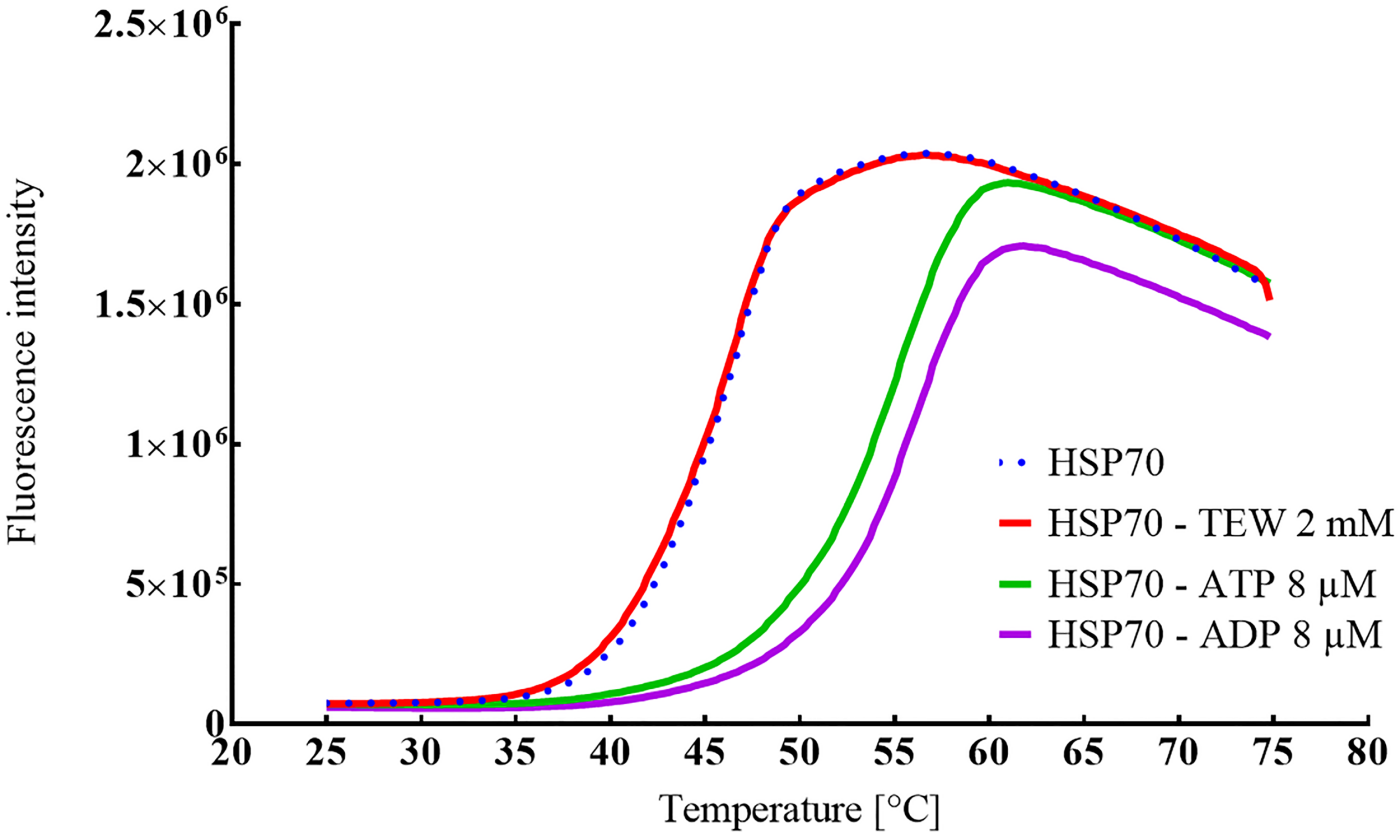
Thermal unfolding (DSF) curves of HSP70 alone and in the presence of ligands. HSP70 (Tm = 45.23±0.13 K) and combined with ligands TEW (Tm = 44.84 ± 0.33 K), ADP (Tm = 54.48 ± 0.34 K) or ATP (Tm = 53.3 ± 0.24 K). Lines represent the mean of quadruplicate fluorescence intensity measurements; error bars have been omitted for clarity.

The shape of HSP70 thermal unfolding curves (Fig 8) with and without the natural ligands ATP (delta Tm 8.31 K at 8 μM) and ADP (deltaTm 9.2 K at 8 μM) indicate that the thermal denaturation process is reliably detected. The stability curves of HSP70 in absence and in presence of up to 2mM TEW were similar, indicating no stabilizing effect.

## Discussion

This study adds one further example of the influence of TEW on protein crystallization and highlights its broad applicability as a crystallization additive. The addition of TEW to HSP70 resulted in an additional crystal packing (already reported for a single HSP70 inhibitor complex) compared to an identical screen in the absence of TEW. Although the addition of TEW did not give rise to a previously unobserved crystal packing, it significantly altered the crystallization behavior of HSP70 in our crystallization screens. Based on these observations and the fact that two TEW molecules are bound at a single crystal packing interface unique to crystal form 2, we propose that under our screening conditions the crystallization of HSP70 in this crystal form is favored by the presence of TEW.

The TEW molecules bound to two different locations on the HSP70 surface with split occupancy due to static and/or dynamic translational disorders of the TEW molecules in the crystal. It is possible that many low affinity ligands exhibit similar “diffuse” binding modes and that only the strong scattering power of the heavy atoms of TEW, together with its rigidity and distinctive shape, allows its observation in the crystal structure. In this limited study, crystallization screening was carried out using a single concentration of TEW. It is possible that screening at several TEW concentrations and/or combining this additive with other methods such as matrix microseeding would result in the identification of additional new crystal forms.

We believe that TEW and related additives such as the “crystallophore” terbium complexes [30] will be important tools for the crystallization of difficult protein targets as well as the generation of crystals suitable for inhibitor soaking. The identification of additional small molecules with varying shape, rigidity and surface charge properties that can enhance protein crystallization in a similar way to TEW would be of great benefit, not only for individual crystallization projects but to help us to learn more about the properties of broadly applicable crystallization additives and how they facilitate crystallization.

## Supporting information

S1 Fig Anomalous Fo-Fc omit map of TEW in crystal form 1, contoured at 5 sigma (1.4 Å resolution). (TIF)

S2 Fig Anomalous Fo-Fc omit map of TEW in crystal form 2, site 1, contoured at 5 sigma (2.3 Å resolution). (TIF)

S3 Fig Anomalous Fo-Fc omit map of tentatively modelled TEW in crystal form 2, site 2, contoured at 2 sigma (white) and 3 sigma (green) (2.3 Å resolution). (TIF)

S4 Fig Fo-Fc omit map (non-anomalous) of tentatively modelled TEW in crystal form 2, site 2, contoured at 3 sigma (2.3 Å resolution). (TIF)

S5 Fig Overlay of HSP70 NBD crystal form 2 (grey, ADP and TEW shown as sticks) with PDB entry 1HJO (magenta). (PNG)

S6 Fig TEW bound crystal form 2, both TEW molecules bind at a single crystal contact. (TIF)
