# Supplementary figures and images for: The crystallization additive hexatungstotellurate promotes the crystallization of the HSP70 nucleotide binding domain into two different crystal forms
Source: PLoS One. 2018 Jun 27;13(6):e0199639. doi: 10.1371/journal.pone.0199639 (PMC6021075; doi:10.1371/journal.pone.0199639)

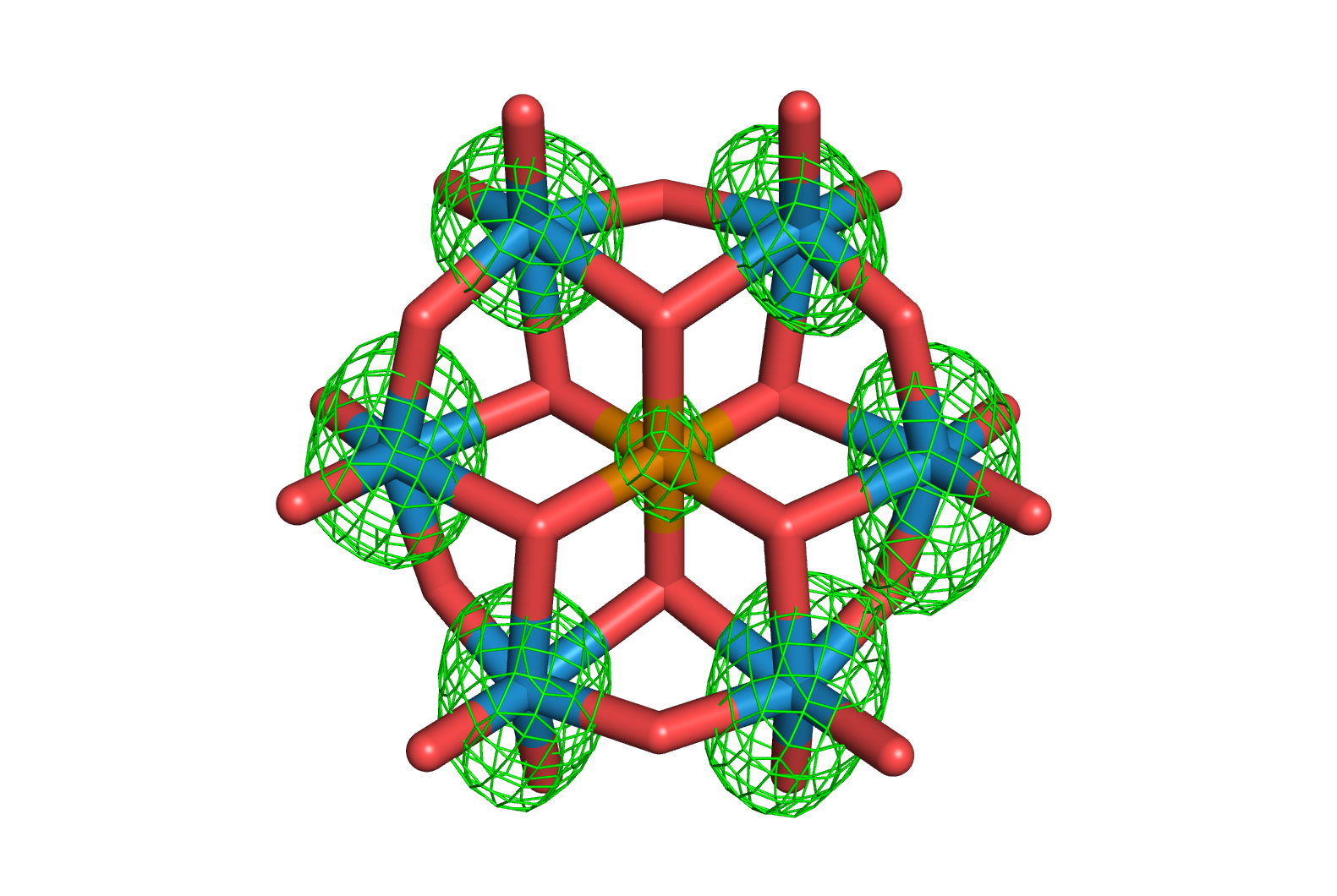

Supplement: S1 Fig — (TIF) [file pone.0199639.s001.tif]

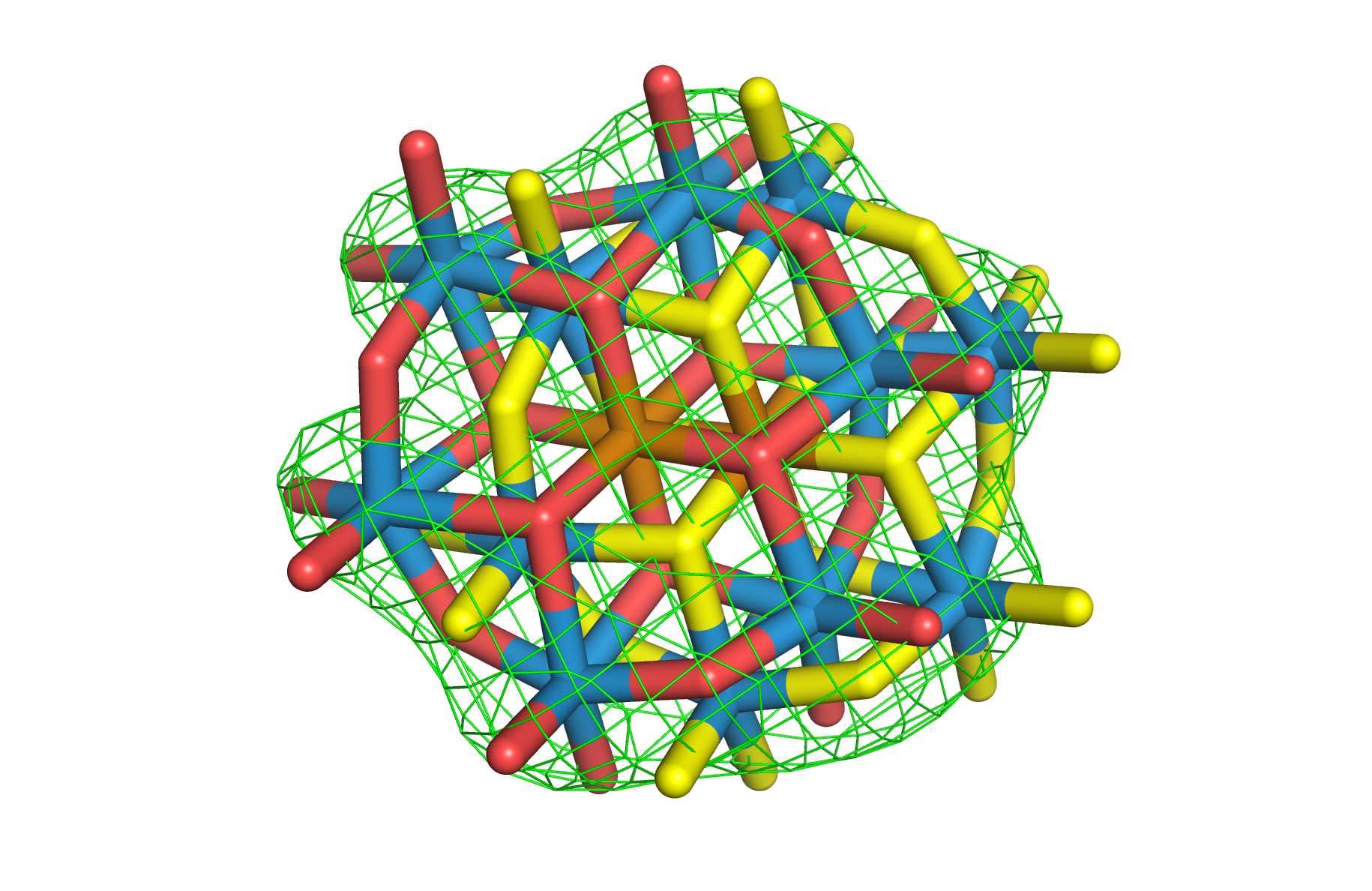

Supplement: S2 Fig — (TIF) [file pone.0199639.s002.tif]

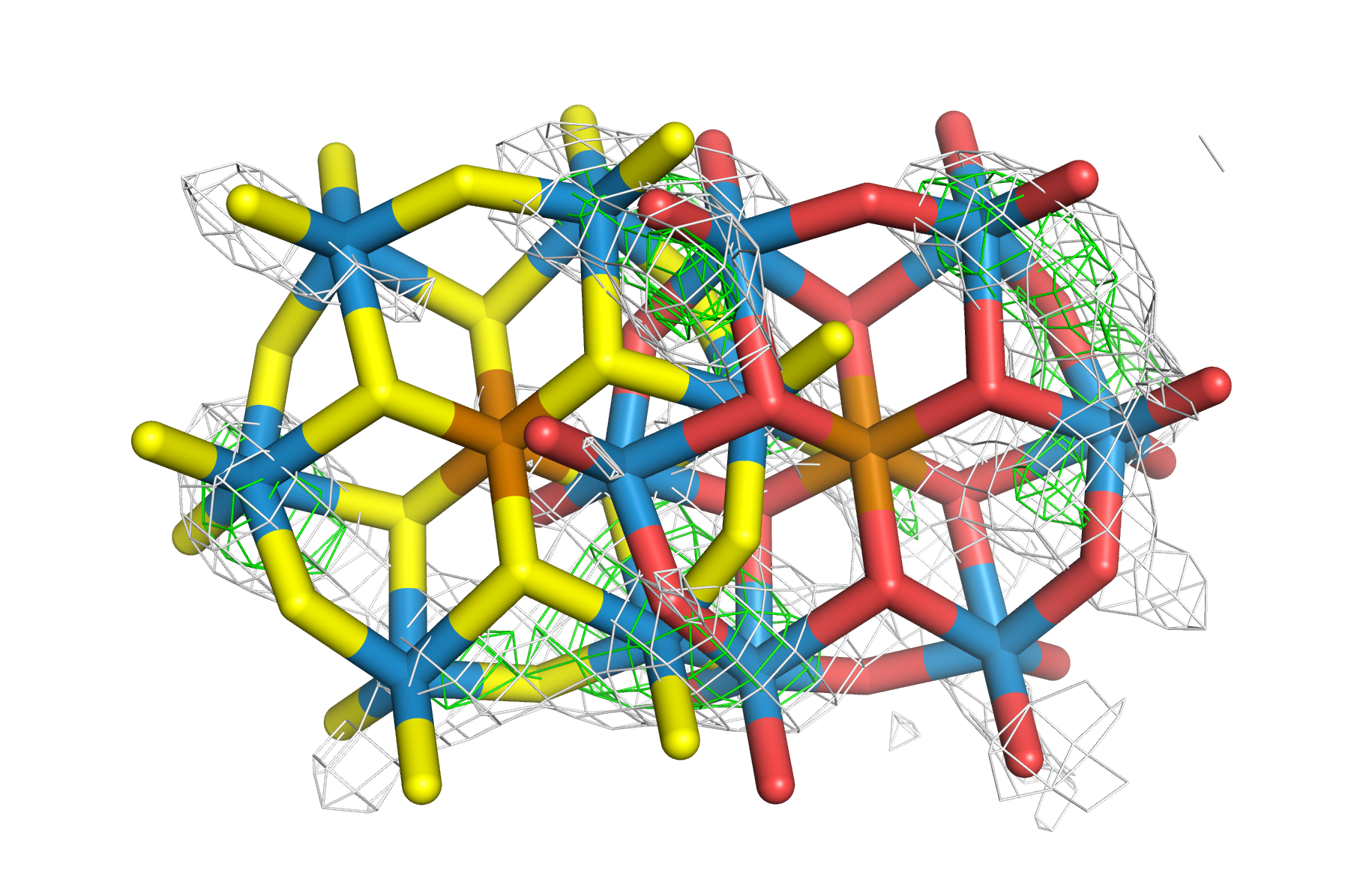

Supplement: S3 Fig — (TIF) [file pone.0199639.s003.tif]

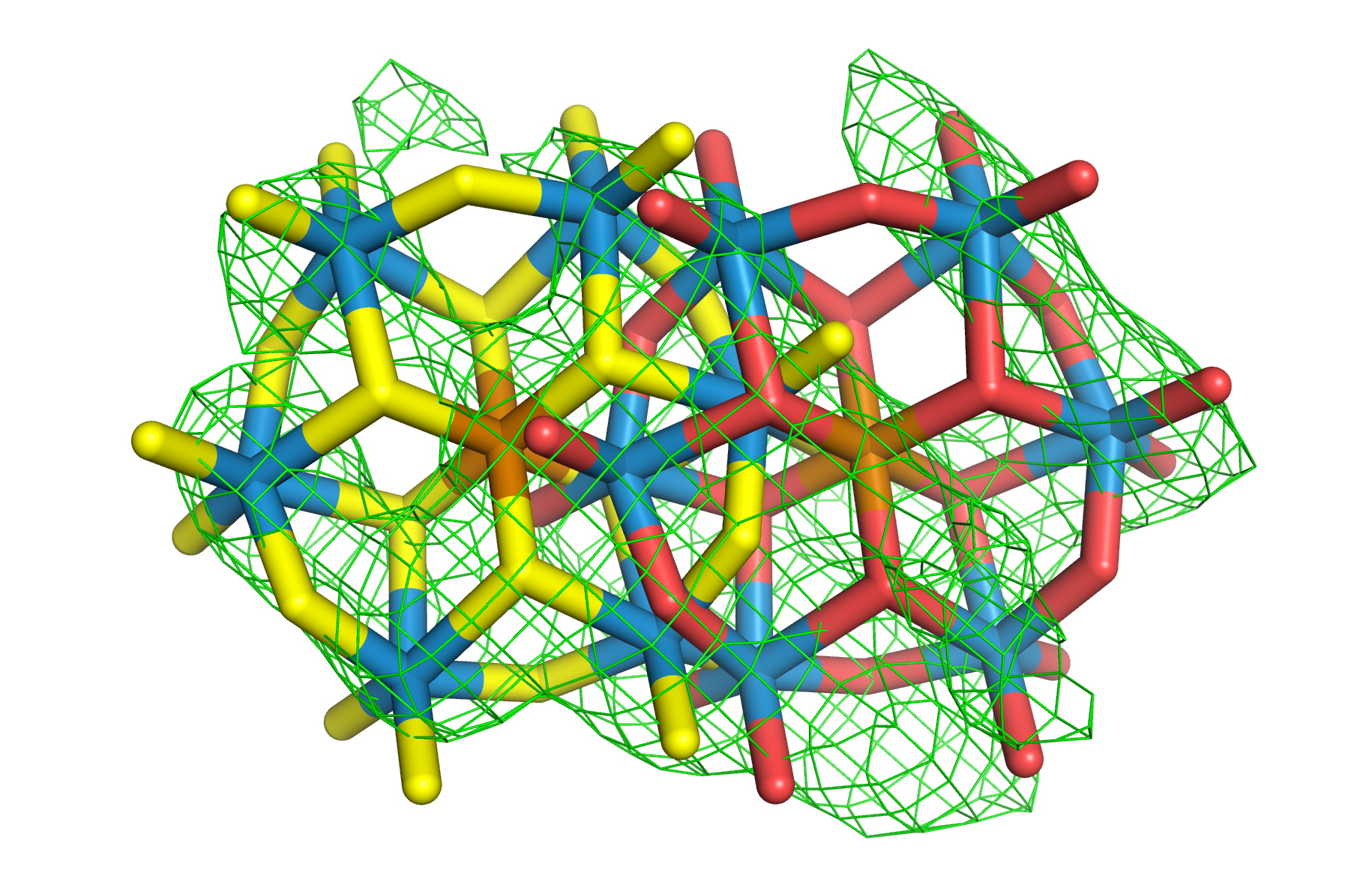

Supplement: S4 Fig — (TIF) [file pone.0199639.s004.tif]

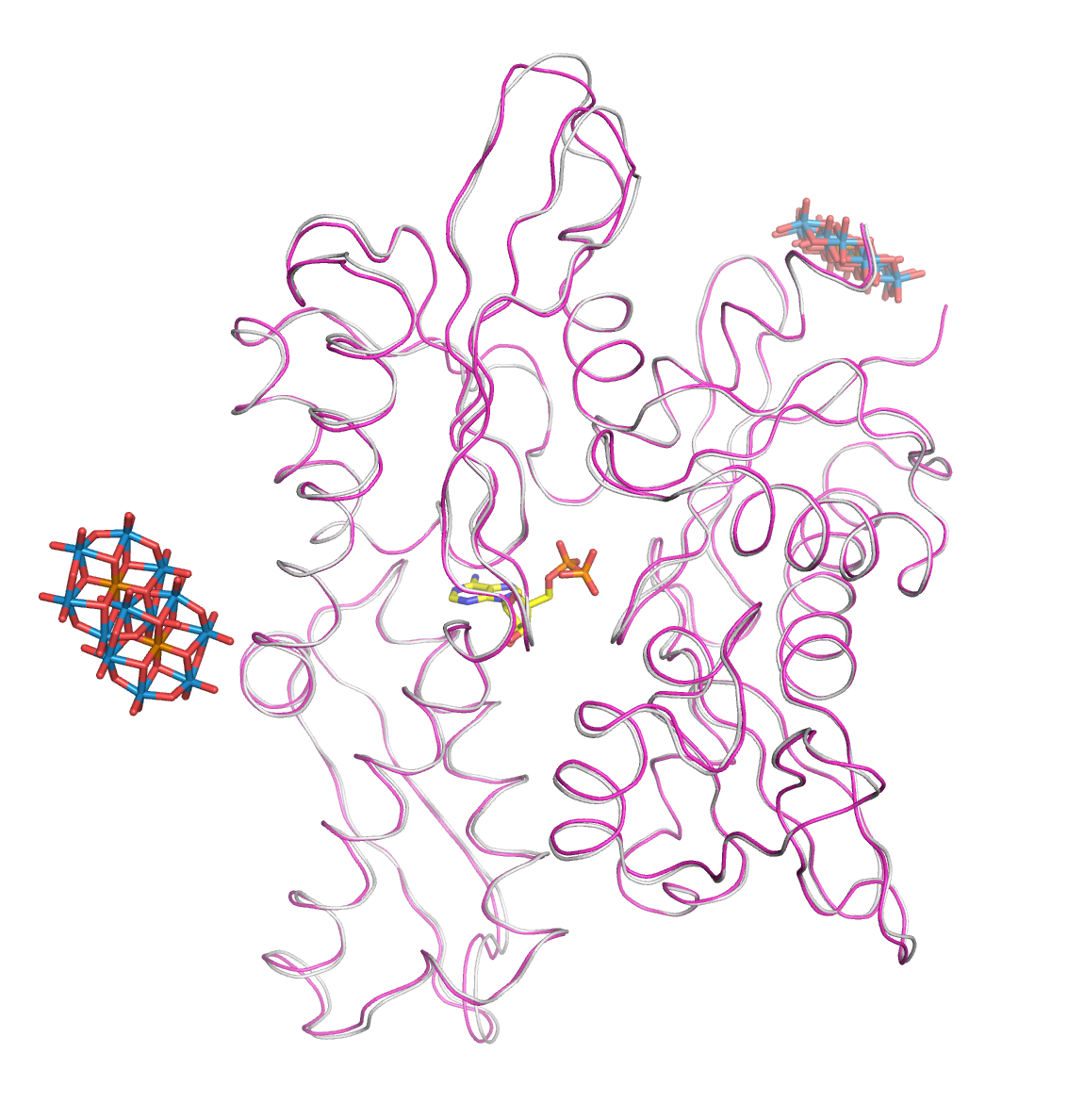

Supplement: S5 Fig — (PNG) [file pone.0199639.s005.png]

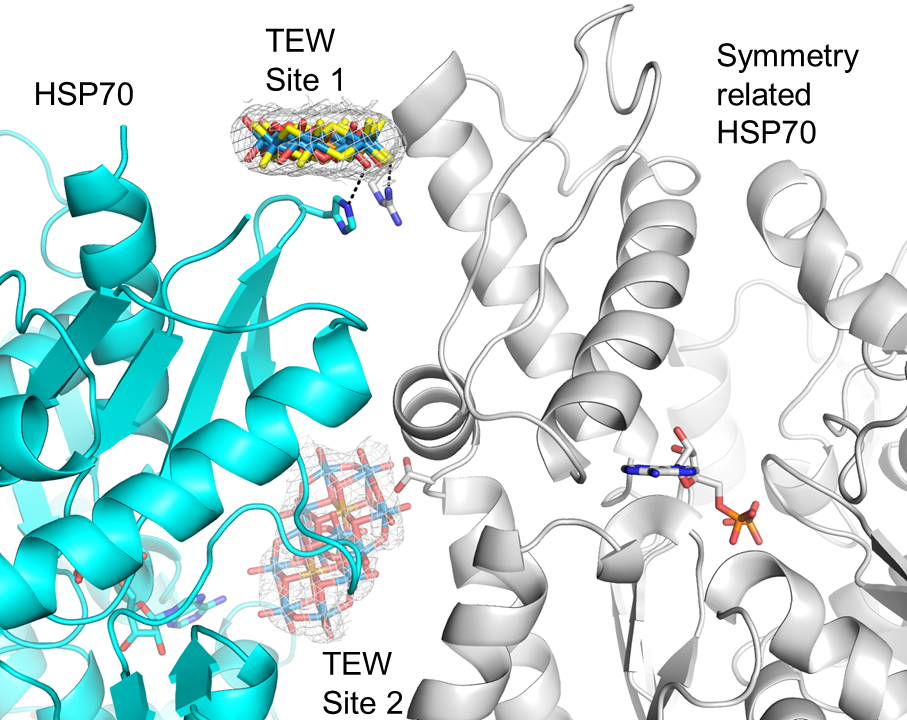

Supplement: S6 Fig — (TIF) [file pone.0199639.s006.tif]
